# Effect of whole-brain radiotherapy with platinum-based chemotherapy in non-small cell lung cancer patients with multiple metastases including brain metastases

**DOI:** 10.1038/s41598-023-40235-0

**Published:** 2023-08-14

**Authors:** Woo Kyung Ryu, Hyung Keun Cha, Woochul Kim, Ha Young Lee, Hyun-Jung Kim, Jeong-Seon Ryu, Jun Hyeok Lim

**Affiliations:** 1 Center for Lung Cancer, Division of Pulmonology, Department of Internal Medicine, Inha University Hospital, Inha University College of Medicine, 27, Inhang-Ro, Jung-Gu, Inchon, 22332 Republic of Korea; 2 Department of Radiation Oncology, Inha University Hospital, Inha University College of Medicine, Inchon, Republic of Korea; 3 Department of Radiology, Inha University Hospital, Inha University College of Medicine, Inchon, Republic of Korea

**Keywords:** Non-small-cell lung cancer, Metastasis

## Abstract

Current guidelines recommend that cytotoxic chemotherapy be considered first in non-small cell lung cancer (NSCLC) patients with multiple metastases, and whole-brain radiotherapy (WBRT) is not initially recommended even if brain metastases are present. However, cytotoxic chemotherapeutic agents are less effective in brain metastases due to poor blood–brain barrier permeability. We investigated the effect of WBRT in combination with cytotoxic chemotherapy on survival in NSCLC patients who were EGFR, ALK, and PD-L1 negative, had an ECOG PS of 2, and had multiple metastases including brain metastases. From January 2005 to December 2018, histologically confirmed NSCLC patients who were EGFR, ALK, and PD-L1 negative, had an ECOG PS of 2, and had multiple metastases including brain metastases were included in this study. Patients were classified into two groups based on receiving WBRT prior to or concurrently with administration of first-line chemotherapeutic agents or receiving chemotherapy only. We compared intracranial progression-free survival (iPFS) and overall survival (OS). Of the 240 NSCLC patients with brain metastases at diagnosis and an ECOG PS of 2, 67 patients were EGFR, ALK, and PD-L1 negative with multiple metastases including brain metastases. Among those patients, 43 (64.2%) received WBRT prior to or concurrently with platinum-based chemotherapy. Patients who received WBRT prior to or concurrently with chemotherapy had better iPFS (7.7 months [4.8–10.6] vs. 3.5 months [2.1–4.9], p = 0.009) and OS (10.8 months [5.9–15.7] vs. 6.1 months [1.9–10.3], p = 0.038) than those who did not receive WBRT. In multivariate analyses, WBRT was significantly associated with iPFS (HR: 1.94 and 95% CI 1.11–3.40, p = 0.020) and OS (HR: 1.92 and 95% CI 1.08–3.42, p = 0.027). In NSCLC patients who are EGFR, ALK, and PD-L1 negative, have an ECOG PS of 2, and have multiple metastases including brain metastases, WBRT prior to or concurrently with chemotherapy could improve iPFS and OS. Therefore, the combination of WBRT with cytotoxic chemotherapy should be considered in these patients.

## Introduction

Non-small cell lung cancer (NSCLC) with brain metastases is the most common central nervous system malignancy, accounting for as much as 20% of every brain metastasis case^1^. Patients with NSCLC with brain metastases often have a poor prognosis, with the median survival ranging between 4 and 6 months^2^. Whole-brain radiotherapy (WBRT) is often considered for patients in whom surgery or stereotactic radiosurgery is not recommended. However, it is unclear whether WBRT provides clinically significant benefits for NSCLC patients with brain metastases^3^. Therefore, current guidelines recommend that systemic chemotherapy be considered first in NSCLC patients with multiple metastases, and WBRT is not initially recommended, even if brain metastases are present^4^.

If tumors are negative for druggable molecular markers and are PD-L1 low (< 1%), systemic chemotherapeutic agents, including cytotoxic agents and immune checkpoint inhibitors (ICI), are considered rather than targeted agents. Specifically, only cytotoxic chemotherapeutic agents are recommended in patients with an Eastern Cooperative Oncology Group performance status (ECOG PS) of 2. However, unlike ICI, cytotoxic chemotherapeutic agents are less effective in brain metastases due to poor blood–brain barrier (BBB) permeability^5^. Approximately 30–50% of NSCLC patients are PD-L1 low, and half of those patients are epidermal growth factor receptor (EGFR) and anaplastic lymphoma kinase (ALK) negative^6,7^. Therefore, given the considerable proportion of patients who are recommended to be treated with cytotoxic chemotherapeutic agents alone under the current guidelines, it is necessary to assess whether WBRT can provide a clinically significant benefit for patients with brain metastases.

In this study, we investigated the effect of WBRT in combination with cytotoxic chemotherapy on survival in NSCLC patients who were EGFR, ALK, and PD-L1 negative, had an ECOG PS of 2, and had multiple metastases including brain metastases.

## Methods

### Study population

**Figure 1:**
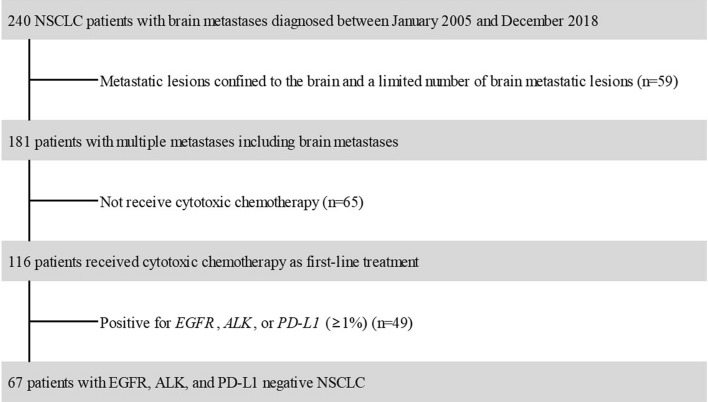
Flowchart of patient selection.

A total of 240 patients with histologically confirmed NSCLC who were diagnosed between January 2005 and December 2018 at Inha University Hospital (Incheon, Republic of Korea) were initially considered for this study (Fig. 1). All patients had an ECOG PS of 2 and had brain metastasis at the time of diagnosis. Patients with metastatic lesions confined to the brain and a limited number of brain metastatic lesions (n = 59) were excluded. The limiting number of metastatic lesions was defined as five or less according to the NCCN guidelines^4^. Patients who did not receive cytotoxic chemotherapy (n = 65) were excluded. Among the remaining patients, those who were positive for EGFR, ALK, or PD-L1 (≥ 1%) (n = 49) were excluded. In total, 67 NSCLC patients who were EGFR, ALK, and PD-L1 negative, had an ECOG PS of 2, and had multiple metastases including brain metastases were included in this study.

Information such as age, gender, smoking history, histology, organs of metastasis, and chemotherapeutic agents were analyzed. The stage of all patients was estimated according to the eighth edition of the TNM classification system^8^. Volumes of all lesions were calculated using linear dimensions of the tumors (CC, LR, AP) obtained from pretreatment imaging tests. Considering the fact that brain metastases usually have a sphere-like shape, the formula for the volume of a spheroid: 4/3 × π × (CC/2) × (LR/2) × (AP/2) was used to simplify volume assessment^9,10^. All patients received platinum-based chemotherapy as the first line chemotherapeutic agent up to six cycles unless patients underwent disease progression or death. All information was collected prospectively from electronic medical records of the Lung Cancer Cohort of Inha University Hospital^11^. This study was approved by the Institutional Review Board of Inha University Hospital. The need for informed consent was waived by Institutional Review Board of Inha University Hospital (IRB number: 2021-07-032).

### Brain radiotherapy and response evaluation

Patients were classified into two groups based on whether they received WBRT. WBRT was delivered prior to or concurrently with administration of first-line chemotherapeutic agents. For WBRT, three-dimensional conventional radiotherapy was done by a linear accelerator (RapidArc, Varian/Mevatron, Siemens). Patients were received WBRT dose of 30 Gy in 10 fractions using 4–6 MV X-ray. Each patient was treated in the supine position while wearing a head immobilization mask to ensure that daily positioning was reproducible. The ocular lens was shielded from the direct beam at all times using collimators. Hippocampal sparing was not applied. The planning target volume of WBRT was defined as the whole-brain parenchyma plus 5 mm margins as visualized on the simulator or portal films to account for beam penumbra and day-to-day set-up variation. A dosimetrist optimized the multileaf collimator shape used in the treatment based on the CT image in the treatment planning system of Eclipse.

Chest and abdomen computed tomography (CT), 18F-fluorodeoxyglucose positron emission tomography/computed tomography (FDG-PET/CT) or bone scan, and brain magnetic resonance imaging (MRI) were conducted using standard protocols at the time of diagnosis and approximately every 12 weeks thereafter. The response of intracranial lesions to chemotherapy with or without WBRT was evaluated by brain MRI that was included in the tests for the re-evaluation. In patients received WBRT, brain MRI for the first re-evaluation of intracranial lesion was performed about 1–2 months after completion of WBRT. Responses were evaluated using RECIST (Response Evaluation Criteria in Solid Tumors) 1.1 criteria^12^. The objective response rate (ORR) of intracranial lesions included the combination of complete response (CR) and partial response (PR), and the disease control rate (DCR) of intracranial lesions included CR, PR, and stable disease (SD).

### Statistical analysis

To assess the association between WBRT and clinical variables, Pearson’s chi-square tests were conducted. Mann Whitney U-test was used to compared the number and volume of brain metastases between WBRT group and non-WBRT group. Overall survival (OS) was defined as the time from the start of chemotherapy to death. Intracranial progression-free survival (iPFS) was defined as the time from the start of chemotherapy to radiologically confirmed intracranial progression or death. The Kaplan–Meier method was adopted to estimate OS and iPFS, and survival curves were compared using log-rank tests. To assess the effect of WBRT, we performed univariate and multivariate analyses using the Cox proportional hazards model. Variables that had a value of p ≤ 0.1 in univariate analyses were included in a multivariate Cox proportional hazards model. Statistical significance was considered as two-sided p values ≤ 0.05. All statistical analyses were performed by using a statistical software package (SPSS, version 19.0).

### Ethics approval and consent to participate

This study was approved by the Institutional Review Board of Inha University Hospital. The need for informed consent was waived by Institutional Review Board of Inha University Hospital (IRB number: 2021-07-032). All methods were performed in accordance with relevant guidelines and regulations.

## Results

### Patient characteristics

In total, 67 NSCLC patients who were EGFR, ALK, and PD-L1 negative, had an ECOG PS of 2, and had multiple metastases including brain metastases received a combination of platinum-based chemotherapy as first-line treatment. Among these patients, 43 patients received WBRT prior to or concurrently with administration of first-line chemotherapeutic agents. Of the 43 patients who underwent WBRT, 7 patients (16.3%) received WBRT concurrently with chemotherapy and 36 patients (83.7%) prior to chemotherapy. Table 1 summarizes the baseline characteristics of the study population. The median follow-up time was 7.4 months (range 0.7–69.6 months). The median age of the patients was 65 years (range 34–81 years). Patients in the WBRT group had a statistically significant higher number (median value and interquartile range [IQR], 4 and 1–10 in WBRT group vs. 1 and 1–4 in non-WBRT group, p = 0.014) and larger volume (median value and IQR, 2.3 and 0.3–8.4 in WBRT group vs. 0.2 and 0.1–0.7 in non-WBRT group, p < 0.001) of brain metastases than those in the non-WBRT group. Meanwhile, there was no significant difference in the proportion of patients with neurological symptoms^13^ between the two groups (25.6% in WBRT group vs. 12.5% in non-WBRT group, p = 0.207). Except for the number and volume of brain metastases, no statistically significant differences were found in the baseline characteristics between the two groups.

**Table 1:** Baseline characteristics of the study population.

Variables	All patients (n = 67)	WBRT		
		Yes (n = 43)	No (n = 24)	p value
Age				
< 65	32 (47.8)	23 (53.5)	9 (37.5)	0.209
≥ 65	35 (52.2)	20 (46.5)	15 (62.5)	
Gender				
Male	45 (67.2)	30 (69.8)	15 (62.5)	0.544
Female	22 (32.8)	13 (30.2)	9 (37.5)	
Smoking history				
Never	26 (38.8)	16 (37.2)	10 (41.7)	0.720
Ever	41 (61.2)	27 (62.8)	14 (58.3)	
Histology				
SQC	8 (11.9)	5 (11.6)	3 (12.5)	0.305
ADC	55 (82.1)	34 (79.1)	21 (87.5)	
Other	4 (6.0)	4 (9.3)	0 (0.0)	
Number of metastatic organs				
1 (brain)	4 (6.0)	3 (7.0)	1 (4.2)	0.719
2	18 (26.9)	12 (27.9)	6 (25.0)	
3	17 (25.4)	12 (27.9)	5 (20.8)	
4	19 (28.4)	12 (27.9)	7 (29.2)	
5 or more	9 (13.4)	4 (9.3)	5 (20.8)	
Number of brain metastases, median (IQR)	2 (1–8)	4 (1–10)	1 (1–4)	0.014
Volume of brain metastases (cm^3^), median (IQR)	0.8 (0.1–6.5)	2.3 (0.3–8.4)	0.2 (0.1–0.7)	< 0.001
Chemotherapy agent				
Irinotecan/cisplatin	18 (26.9)	8 (18.6)	10 (41.7)	0.187
Gemcitabine/cisplatin	18 (26.9)	14 (32.6)	4 (16.7)	
Pemetrexed/cisplatin	23 (34.3)	16 (37.2)	7 (29.1)	
Other	8 (11.9)	5 (11.6)	3 (12.5)	

*WBRT* whole-brain radiotherapy, *SQC* squamous cell carcinoma, *ADC* adenocarcinoma, *IQR* interquartile range.

### Intracranial treatment response

Intracranial responses could be evaluated by brain MRI in 55 patients (82.1%). In the other patients (n = 12), follow-up of intracranial lesions by brain MRI was not performed due to their short OS. Among the 37 patients in the WBRT group, assessment of intracranial lesions indicated that 1 patient (2.7%) had CR, 22 patients (59.5%) had PR, 11 patients (29.7%) had SD, and 3 patients (8.1%) had progressive disease (PD). Whereas 1 patient (5.6%) had PR, 10 patients (55.6%) had SD, and 7 patients (38.9%) had PD among the 18 patients in the non-WBRT group. In the non-WBRT group, 6 of 7 patients with intracranial PD received WBRT after confirmation of intracranial PD. Intracranial ORR was 62.2% for the WBRT group and only 5.6% for the non-WBRT group (p < 0.001). Intracranial DCR was 91.9% for the WBRT group and 61.1% for the non-WBRT group (p = 0.005).

### Survival following whole-brain radiotherapy

**Figure 2:**
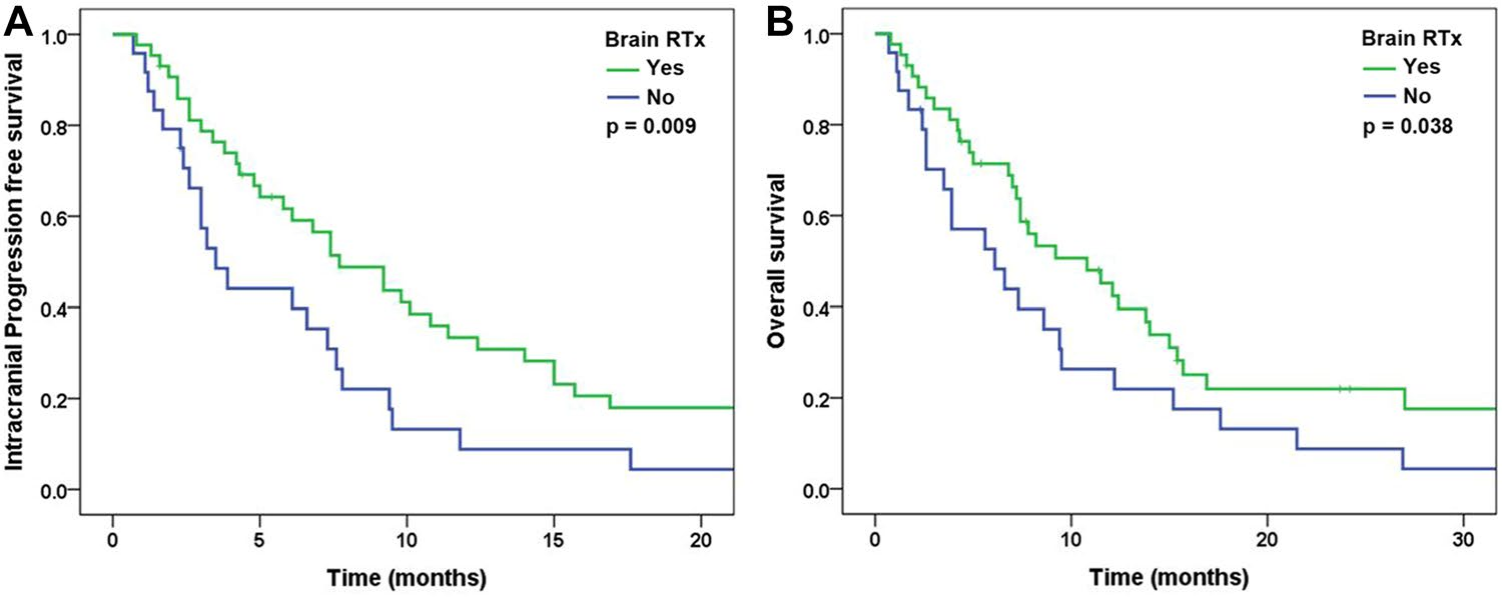
Survival by whole-brain radiotherapy prior to or concurrent with chemotherapy in non-small-cell lung cancer patients who were EGFR, ALK, and PD-L1 negative, had an ECOG PS of 2, and had multiple metastases including brain metastases. (**A**) Intracranial progression-free survival. (**B**) Overall survival.

The median iPFS and OS times were 6.8 months (95% CI 5.1–8.5 months) and 8.2 months (95% CI 6.0–10.4 months). Patients who received WBRT prior to or concurrently with chemotherapy had better iPFS (7.7 months [4.8–10.6] vs. 3.5 months [2.1–4.9], p = 0.009) and OS (10.8 months [5.9–15.7] vs. 6.1 months [1.9–10.3], p = 0.038) than those who did not receive WBRT (Fig. 2). In multivariate analyses, WBRT was significantly associated with iPFS (HR: 1.94 and 95% CI 1.11–3.40, p = 0.020) and OS (HR: 1.92 and 95% CI 1.08–3.42, p = 0.027) (Table 2).

**Table 2:** Effect of whole-brain radiotherapy prior to or concurrent with chemotherapy on survival.

Variables	Intracranial progression-free survival				Overall survival			
	Univariate analysis		Multivariate analysis		Univariate analysis		Multivariate analysis	
	HR (95% CI)	p value	HR (95% CI)	p value	HR (95% CI)	p value	HR (95% CI)	p value
Age		0.341				0.051		0.377
< 65	Reference				Reference		Reference	
≥ 65	1.29 (0.77–2.15)				1.72 (1.00–2.95)		1.31 (0.72–2.40)	
Gender		0.111				0.051		0.147
Male	Reference				Reference		Reference	
Female	0.63 (0.36–1.11)				0.57 (0.32–1.00)		0.62 (0.32–1.19)	
Smoking history		0.511				0.538		
Never	Reference				Reference			
Ever	1.19 (0.71–2.02)				1.18 (0.69–2.02)			
Histology		0.529				0.494		
SQC	Reference				Reference			
ADC	0.76 (0.30–1.91)				0.69 (0.27–1.75)			
Others	1.29 (0.34–4.86)				1.14 (0.31–4.30)			
Number of metastatic organs		0.004		0.008		0.008		0.097
1 (brain)	Reference		Reference		Reference		Reference	
2	1.92 (0.64–5.78)		2.04 (0.67–6.19)		1.84 (0.53–6.39)		1.75 (0.48–6.35)	
3	1.45 (0.48–4.39)		1.46 (0.48–4.42)		1.74 (0.50–6.04)		1.64 (0.46–5.82)	
4	0.82 (0.26–2.58)		0.81 (0.26–2.57)		0.84 (0.23–3.07)		0.98 (0.26–3.69)	
5 or more	4.62 (1.38–15.44)		4.08 (1.20–13.80)		4.45 (1.18–16.74)		3.66 (0.94–14.25)	
Number of brain metastases	1.00 (0.98–1.03)	0.783			1.00 (0.97–1.03)	0.949		
Volume of brain metastases	0.99 (0.97–1.01)	0.269			0.99 (0.97–1.01)	0.411		
Chemotherapy agent		0.606				0.774		
Irinotecan/cisplatin	Reference				Reference			
Gemcitabine/cisplatin	0.65 (0.32–1.33)				0.92 (0.44–1.93)			
Pemetrexed/cisplatin	0.89 (0.46–1.71)				1.30 (0.66–2.59)			
Others	0.66 (0.27–1.61)				0.97 (0.40–2.36)			
Brain RTx		0.010		0.020		0.041		0.027
Yes	Reference		Reference		Reference		Reference	
No	2.02 (1.18–3.47)		1.94 (1.11–3.40)		1.76 (1.02–3.02)		1.92 (1.08–3.42)	

*HR* hazards ratio, *CI* confidence interval, *SQC* squamous cell carcinoma, *ADC* adenocarcinoma, *RTx* radiotherapy.

### A representative case

**Figure 3:**
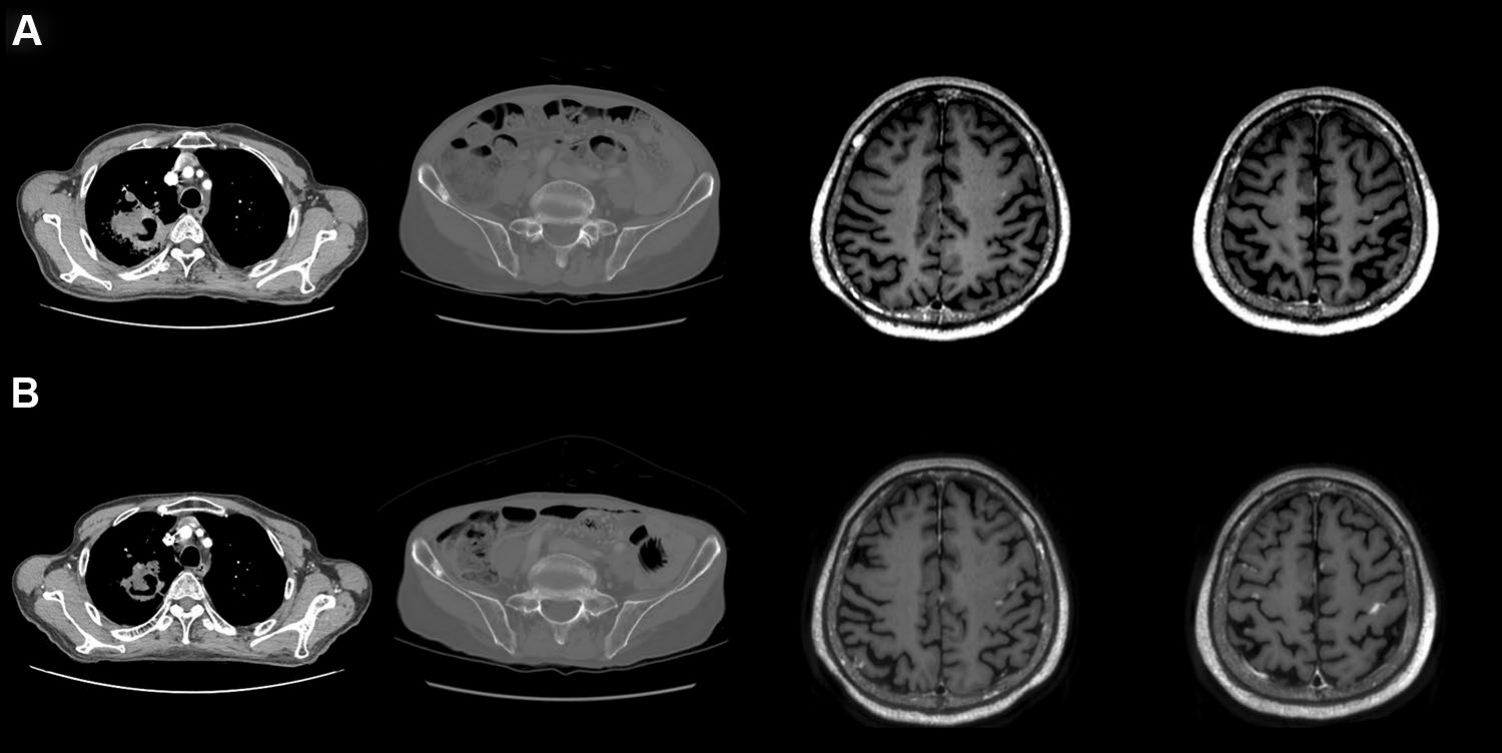
A typical case with brain metastases that showed disease progression after receiving only chemotherapy without whole-brain radiotherapy. Compared to the time of lung cancer diagnosis (**A**), the size of the primary tumor decreased and that of the metastatic lesion in the right iliac bone did not change after two cycles of platinum-based chemotherapy (**B**). However, the size of metastatic lesions in the brain increased and new metastatic lesions developed.

A representative case with brain metastases that showed disease progression after receiving only chemotherapy without WBRT is highlighted in Fig. 3. A 75-year-old man was diagnosed with stage IV lung adenocarcinoma with metastasis in the right iliac bone and multiple metastases in the brain. The patient complained of right hemiplegia after 2 cycles of platinum-based chemotherapy. As indicated by imaging examination, the primary tumor decreased in size, and the metastatic lesion in the right iliac bone did not change; however, the metastatic lesions in the brain increased in size and new metastatic lesions developed. The patient died 4 months after lung cancer diagnosis due to rapid deterioration of general health conditions associated with aggravated neurologic symptoms.

## Discussion

In this study, patients who received WBRT prior to or concurrently with chemotherapy had improved intracranial ORR and intracranial DCR compared to those who did not receive WBRT. In addition, the WBRT group had better iPFS and OS than the non-WBRT group. These results suggest that combining WBRT with cytotoxic chemotherapy could be beneficial for survival in NSCLC patients who are EGFR, ALK, and PD-L1 negative, have an ECOG PS of 2, and have multiple metastases including brain metastases. Patients in the WBRT group showed better iPFS and OS than the those in the non-WBRT group despite the higher number and larger volume of brain metastases at diagnosis. Also, unlike a previous study^14^, the volume of brain metastases was not significantly associated to iPFS and OS. We suggest that it might be associated to the strong impact of WBRT on survival.

The QUARTZ trial showed that WBRT with supportive care does not significantly affect OS compared to supportive care only^3^. This finding is contrary to the results of our study in that WBRT provided a significant OS benefit. However, in the QUARTZ trial, patients did not receive systemic chemotherapy, so a significant proportion of patients did not live long enough to experience the effects of WBRT unlike in this study; the maximum benefit of WBRT is achieved approximately 6 weeks after the end of treatment^15^.

The BBB is an obstacle for effective chemotherapies for brain metastases, thus suggesting that brain radiotherapy will continue to play a role in the management of brain metastases^16^. Furthermore, previous studies have provided evidence that brain radiotherapy disrupts the BBB, allowing chemotherapeutic agents to better reach brain metastases^17,18^. These findings are supported by our results in that WBRT prior to or concurrently with chemotherapy provides survival benefits.

In previous studies, patients with poor performance, such as those with Karnofsky Performance Scores (KPS) < 70 or ECOG PS ≥ 2, showed no benefit from WBRT^3,19,20^. Although a significant number of patients were included in the PS = 2 group of the previous studies, the survival benefit of WBRT in patients with PS = 2 was not determined as the patients with PS = 2 were grouped with patients with PS 3 or 4^21,22^. In this study, we considered the clinical significance of patients with PS = 2, and only patients with PS = 2 were included in this study. The significant benefit of WBRT plus cytotoxic chemotherapy was demonstrated in the PS = 2 group.

There are limitations of this study. First, this study was conducted at a single center and external confirmatory studies should be conducted in the future. However, all clinical information was obtained prospectively from the Inha Lung Cancer Cohort. Second, although it is important to consider the quality of life and the toxicity of treatment, these considerations could not be investigated in this study^23^. Considering that not only WBRT but also chemotherapy can affect cognitive function^24,25^, it is necessary to investigate changes in cognitive function before and after treatment in the WBRT with chemotherapy group and chemotherapy alone group in the future. Third, because of the small number of patients, it was difficult to conduct a subgroup analysis such as dividing into patients received WBRT concurrently with chemotherapy and those received WBRT prior to with chemotherapy. We believe that a study with a larger number of patients is needed in the future.

## Conclusions

WBRT prior to or concurrently with chemotherapy improved iPFS and OS in NSCLC patients who were EGFR, ALK, and PD-L1 negative, had an ECOG PS of 2, and had multiple metastases including brain metastases. Therefore, we suggest that the combination of WBRT with cytotoxic chemotherapy should be considered in these patients. The combination of WBRT with cytotoxic chemotherapy should be carefully individualized for each patient with consideration of each patient’s situation, such as characteristics of metastatic lesions and general health conditions.

## Data Availability

The datasets used and/or analysed during the current study are available from the corresponding author on reasonable request.

## Abbreviations

NSCLC: Non-small cell lung cancer
WBRT: Whole-brain radiotherapy
ECOG PS: Eastern Cooperative Oncology Group performance status
BBB: Blood–brain barrier
EGFR: Epidermal growth factor receptor
ALK: Anaplastic lymphoma kinase
CT: Computed tomography
FDG-PET/CT: ^18^F-fluorodeoxyglucose positron emission tomography/computed tomography
MRI: Magnetic resonance imaging
RECIST: Response evaluation criteria in solid tumors
ORR: Objective response rate
CR: Complete response
PR: Partial response
DCR: Disease control rate
SD: Stable disease
PD: Progressive disease
OS: Overall survival
iPFS: Intracranial progression-free survival
CI: Confidence interval
HR: Hazard ratio
